# Efficacy of a coating composed of chitosan from *Mucor circinelloides* and carvacrol to control *Aspergillus flavus* and the quality of cherry tomato fruits

**DOI:** 10.3389/fmicb.2015.00732

**Published:** 2015-07-20

**Authors:** Evandro L. de Souza, Camila V. Sales, Carlos E. V. de Oliveira, Laênia A. A. Lopes, Maria L. da Conceição, Lúcia R. R. Berger, Thayza C. M. Stamford

**Affiliations:** ^1^Laboratório de Microbiologia de Alimentos, Departamento de Nuttrição, Centro de Ciências da Saúde, Universidade Federal da ParaíbaJoão Pessoa, Brazil; ^2^Laboratório de Microbiologia, Departamento de Medicina Tropical, Centro de Ciências da Saúde, Unversidade Federal de PernambucoRecife, Brazil

**Keywords:** *Aspergillus*, *Lycopersicon esculentum* Mill, fungi chitosan, phenolic compound, postharvest treatment

## Abstract

Cherry tomato (*Lycopersicon esculentum* Mill) fruits are susceptible to contamination by *Aspergillus flavus*, which may cause the development of fruit rot and significant postharvest losses. Currently there are significant drawbacks for the use of synthetic fungicides to control pathogenic fungi in tomato fruits, and it has increased the interest in exploring new alternatives to control the occurrence of fungal infections in these fruits. This study evaluated the efficacy of chitosan (CHI) from *Mucor circinelloides* in combination with carvacrol (CAR) in inhibiting *A. flavus* in laboratory media and as a coating on cherry tomato fruits (25°C, 12 days and 12°C, 24 days). During a period of storage, the effect of coatings composed of CHI and CAR on autochthonous microflora, as well as on some quality characteristics of the fruits such as weight loss, color, firmness, soluble solids, and titratable acidity was evaluated. CHI and CAR displayed MIC valuesof 7.5 mg/mL and 10 μL/mL, respectively, against *A. flavus*. The combined application of CHI (7.5 or 3.75 mg/mL) and CAR (5 or 2.5 μL/mL) strongly inhibited the mycelial growth and spore germination of *A. flavus*. The coating composed of CHI (3.75 mg/mL) and CAR (2.5 or 1.25 μL/mL) inhibited the growth of *A. flavus* in artificially contaminated fruits, as well as the native fungal microflora of the fruits stored at room or low temperature. The application of the tested coatings preserved the quality of cherry tomato fruits as measured by some physicochemical attributes. From this, composite coatings containing CHI and CAR offer a promising alternative to control postharvest infection caused by *A. flavus* or native fungal microflora in fresh cherry tomato fruits without negatively affecting their quality over storage.

## Introduction

In recent years, the population has shown more interest in acquiring a good quality of life including a balanced diet, which requires a regular consumption of fresh fruits and vegetables. Cherry tomato (*Lycopersicon esculentum* Mill) fruits are of great importance in many countries for economic purposes (Guo et al., 2014; Quiao et al., 2015), with a global production of fresh tomato fruits near 35,000 tons/year. The global consumption of tomato products is near 31,000 tons/year, with an average annual per capita consumption of ∼6 kg/inhabitant and fresh fruits represent near 75% of this consumption (World Processing Tomato Council [WPTC], 2010). However, these fruits are susceptible to various forms of contamination by phytopathogen agents during production, handling, distribution and storage. This contamination can limit their shelf life of tomato fruits and cause postharvest losses as high as 40% in less industrialized countries (Liu et al., 2007; Ramos-García et al., 2012).

*Aspergillus flavus* is among the most common pathogenic fungi affecting cherry tomato fruits, and it grows over the fruit surface, causing losses and decay (*Aspergillus* rot; Tian et al., 2011; Tijjani et al., 2014a,b) and producing an aflatoxin that is a genotoxic, immunotoxic and hepatocarcinogenic secondary metabolite to human beings (Tian et al., 2015). Although synthetic chemical fungicides are the primary means to control populations of pathogenic fungi and related-postharvest diseases in tomato fruits (Sánchez-Domínguez et al., 2011; Chen et al., 2014), their use presents drawbacks in respect to handling hazards, awareness about fungicides residues on food and risks to human health and environment (Soylu et al., 2010). Thus, it has impelled the interest in exploring new alternatives to decrease the use of synthetic fungicides in tomato fruits (Feng et al., 2011). In this context, edible coatings composed of chitosan (CHI) and essential oils or their individual constituents have recently been considered as an environmentally friendly technology to control postharvest decay because of their biodegradability and the lack of phytotoxicity (dos Santos et al., 2012; Beyki et al., 2014).

Chitosan is a deacetylated derivative of chitin. Both CHI and chitin are natural co-polymers, comprising units of 2-amino-2-deoxy-D-glycopyranose and 2-acetamide-2-deoxy-D-glycopyranose interconnected by glycosidic β-1,4 bonds (Coqueiro et al., 2011). The 2-amino-2-deoxy-D-glycopyranose is more frequently observed in CHI (Berger et al., 2014; de Oliveira et al., 2014a). The polymer CHI is a biodegradable non-toxic compound and potential fungicide considered for use in fresh fruits and vegetables due to its biochemical and excellent semi-permeable film-forming properties (Aloui et al., 2014; Chen et al., 2014).

Although the primary commercial source of CHI has been crustacean shells, the fungal production of high-quality CHI by fermentation technology has been heavily exploited due to the great advantages of CHI such as independence from seasonal factors, ability to be produced on a wide scale and simultaneous chitin and CHI extraction (de Oliveira et al., 2014a,b). Moreover, this strategy of producing CHI by fermentation technology avoids protein contamination that could cause allergic reactions in individuals with shellfish allergies (Berger et al., 2014). Obtaining CHI from fungi is known to be a simple process that is economically and environmentally viable, especially when biowaste from an industrial activity, such as production of corn steep liquor (CSL), is used as nutritional source in media culture for cultivation of fungi (Berger et al., 2014). In a previous study, *Mucor circinelloides* (Mucorales order, Zygomycetes class) showed a level of high CHI production when cultivated in CSL-based media (de Oliveira et al., 2014b).

Coatings composed of CHI and other active substances have presented efficacy for inhibiting the growth of phytopathogenic fungi on different fruits (dos Santos et al., 2012; Beyki et al., 2014). Studies have demonstrated strong and wide-spectrum inhibitory effects of different essential oil constituents against food-related pathogenic fungi (Tao et al., 2014). From these studies, the phenolic carvacrol (CAR), primarily related to the antimicrobial property of different essential oils, has emerged as a potent growth inhibitor for different fungi (Feng and Zeng, 2007). However, CAR has still not been rationally exploited as an anti-postharvest pathogenic fungi compound when applied either in free-form or as a component of coating materials. CAR appears to have either no significant or marginal toxic effects *in vivo* and raises no concerns regarding the possible level of use in foods (Pharmaceutical Codex, 1979; Burt, 2004). Although early studies have focused on the antimicrobial activity of crustacean CHI toward different postharvest fungi (Badawy and Rabea, 2009; Ramos-García et al., 2012), there is limited knowledge about the effects of fungal CHI alone or in combination with other antimicrobial compounds on inhibition of postharvest pathogenic fungi in fruits.

Considering these aspects, this study assessed the efficacy of the combined application of CHI from *M. circinelloides* and CAR as a postharvest treatment to prevent *A. flavus* infections in cherry tomato fruits. For this, assays were performed to (1) determine the minimum inhibitory concentration (MIC) of CHI and CAR, (2) evaluate the inhibitory effects of different combinations of these substances at subinhibitory concentrations on *A. flavus* in laboratory media and forming different coatings on cherry tomato fruits and (3) verify the effects of the tested coatings on the physicochemical characteristics of cherry tomato fruits during storage.

## Materials and Methods

### Raw Materials

Cherry tomato (*Lycopersicon esculentum* Mill) fruits were purchased from a local wholesale distributor and selected for uniform characteristics (size, form, appearance and red color) and absence of visible infection signs and/or mechanical injuries (Aloui et al., 2014). Before assays with artificially contaminated fruits and for evaluation of physicochemical quality parameters, the tomato fruits were surface-disinfected by immersion in a sodium hypochlorite solution (150 ppm, pH 7.2 adjusted with 1 M NaOH) for 15 min, washed using sterile distilled water and air-dried for 2 h in a bio-safety cabinet (de Oliveira et al., 2014a,b).

Corn steep liquor used as the substrate for the growth of *M. circinelloides* UCP 050 was obtained from a Brazilian corn processing company (Produtos de Milho, Ltd., Cabo de Santo Agostinho, Brazil). CAR was obtained from Sigma Aldrich (Sigma, France). *M. circinelloides* UCP 050, used for CHI production, was gently supplied from the Culture Collection of the Catholic University of Pernambuco (Recife, Brazil). *A. flavus* URM 4550 was obtained from the University of Recife Mycology Culture Collection (Center for Biological Sciences, Federal University of Pernambuco, Recife, Brazil). This strain was verified for their ability to cause rot in cherry tomato fruits. Continuous procedures of re-inoculations and re-isolations on cherry tomato fruits were performed to maintain the capability of the test *A. flavus* strain to cause fruit rot (Sánchez-Domínguez et al., 2011). For assays, stock cultures were subcultured in Sabouraud agar (Himedia, India) at 28°C for 7 days to reach sufficient sporification (de Oliveira et al., 2014a,b).

### Production of Chitosan

The production of CHI was carried out by submerged cultivation of *M. circinelloides* UCP 050 in a CSL-based medium (7 g CSL/100 mL; pH 5.5 adjusted using 0.1 M HCl, 150 rpm, for 72 h at 25°C) according to a previously described procedure (de Oliveira et al., 2014b). CHI showed a degree of deacetylation of 82% and a characteristic thermal degradation pattern and diffraction peaks (de Oliveira et al., 2014b). The CHI (in powder form) was stored at -20°C.

### Preparation of CHI and CAR Solutions and Coatings

The different CHI solutions were obtained by dissolution of the polymer (20 mg/mL) in acetic acid (1 mL/100 mL, pH 5.6) during 24 h at room temperature (RT) under stirring (120 rpm; dos Santos et al., 2012). Serial dilutions (1:1) were carried out using Sabouraud broth (Himedia, India) to obtain solutions with CHI concentrations varying from 15 to 0.12 mg/mL. To ensure that the observed fungi inhibition was due to the tested CHI and not to acetic acid used in solutions preparation, the pH of all CHI solutions was adjusted to 5.6 using 3 M NaOH. Preliminary experiments showed that the acetic acid solution at pH ≥ 5.6 did not inhibit the growth of the fungi tested strain (de Oliveira et al., 2014a,b). The CAR solutions (10–0.06 μL/mL) were prepared in Sabouraud broth supplemented with bacteriological agar (0.15 g/100 mL; dos Santos et al., 2012).

For the application of CHI and CAR in different combinations, CHI (7.5 or 3.75 mg/mL) was diluted in acetic acid (1 mL/100 mL) for 6 h at RT under stirring (120 rpm). Then, different CAR concentrations (5.0, 2.5, or 1.25 μL/mL) were added and maintained under stirring (120 rpm) for an additional 18 h at RT (dos Santos et al., 2012). To assay the application of the dispersions containing CHI and CAR in combinations as coatings on tomato fruits, glycerol (2 mL/100 mL) was incorporated (simultaneously to the CAR incorporation) as a plasticizer into the coating-forming dispersions (dos Santos et al., 2012).

### Determination of the Minimum Inhibitory Concentration

The MIC of CHI and CAR was determined using broth macrodilution. For this, 1 mL of the fungal spore suspension was inoculated into 4 mL of Sabouraud broth (with the concentration adjusted to 10 mL), and 5 mL of the solutions containing different CHI or CAR concentrations was added (dos Santos et al., 2012; de Sousa et al., 2013). The system was incubated at 28°C for a 7-days period. The lowest CHI or CAR concentration that displayed no visible fungal growth at the end of the incubation period was determined as the MIC (dos Santos et al., 2012).

### Effects on Mycelial Fungal Growth

Inhibition of mycelial growth induced by CHI and CAR was determined using the agar dilution technique. Initially, 10 mL of the *A. flavus* spore suspension (∼10^6^ spores/mL) was dispensed into 90 mL of Sabouraud broth (Himedia, India) containing different concentrations of CHI and CAR, followed for incubation at 25–28°C. After 3- and 7-days incubation period, the mycelial mass was obtained, dried at 60°C for 6 h, and maintained at 40°C for 18 h (de Oliveira et al., 2014a,b). As a control assay, the target fungal suspension was inoculated in Sabouraud broth not containing CHI or CAR. For obtaining the percent inhibition of fungal mycelial growth, the comparison of the weights of the dry mycelial masses obtained from the suspensions exposed to CHI and CAR and from control assay at each of the assessed incubation periods was performed (de Sousa et al., 2013).

### Effects on Fungal Spore Germination

The induction of inhibition in fungal spore germination by CHI and CAR was determined using a broth macrodilution technique. For this, aliquots (0.1 mL) of solutions containing different concentrations of CHI and CAR were mixed with 0.1 mL of the spore suspension (∼10^6^ spores/mL) taken from a fungal culture cultivated for 10 days on Sabouraud agar (Himedia, India) at 25–28°C. Afterward, 0.1 mL of the obtained suspension was placed at the center of a sterile glass slide, followed for incubation at 28°C for 24 h in a moisture chamber. After this period, the slides were fixed with lactophenol cotton blue stain, and the spore germination was observed using light microscopy (dos Santos et al., 2012). Approximately 200 spores were counted on each slide. In the control experiment, CHI and CAR solutions were replaced by Sabouraud broth. The percent inhibition of spore germination was obtained by comparing the number of germinated spores in the media containing CHI and CAR with the number of germinated spores obtained in the control assay (de Oliveira et al., 2014a,b). A spore was considered germinated when developed a germ tube presenting at least twice its original diameter. The results were expressed as percent inhibition rates of spore germination in comparison with the control assay (Aloui et al., 2014).

### Effects On *A. flavus* Infection in Fruits

Cherry tomato fruits were first wounded (3 mm deep and 3 mm wide; four wounds by each fruit; because *A. flavus* invades and causes rot in tomato fruits via wounds in skin of fruits – Snowdon, 1991) with a sterile needle in opposite sides. Thereafter, the fruits were inoculated by immersion in 500 mL of an inoculum solution (∼10^6^ spores/mL) of *A. flavus* and soot-rotated for 1 min using a sterile glass stem, and left to air-dry for 30 min (25°C). Then, the fruits were randomly distributed in different groups and immersed in 500 mL of the tested coating solutions with different CHI and CAR concentrations with slightly shaking for 1 min using a sterile glass stem. The fruits were allowed to air-dry and drain the excess liquid on a nylon-filter and packed in a commercially sterile polyethylene container (with a lid). One group of fruits was maintained at RT [25°C, relative humidity (RH) of 85%], whereas the other was maintained at a low temperature (LT; 12°C, RH of 85%; de Oliveira et al., 2014a,b). Fruits submitted to LT storage were maintained at 12°C because tomato fruits are chilling sensitive at temperatures below 12°C (Tao et al., 2014). As a control treatment, sterile distilled water containing glycerol (2 g/100 mL) were used in replacement of the CHI and CAR dispersions (dos Santos et al., 2012). Each treatment included 40 fruits, which were stored for different intervals (RT for 1, 4, 8, and 12 days; LT for 1, 6, 12, and 24 days). The results were expressed as the storage time for appearance of visible signs of *A. flavus* infection and as the percentage of fruits that presented visible signs of *A. flavus* infection at different time intervals (dos Santos et al., 2012; de Oliveira et al., 2014a,b).

### Effects on Fungal Infection Caused by Native Fungal Microbiota

Cherry tomato fruits (not surface disinfected) were immersed for one min in the solutions containing the CHI and CAR in different concentrations and soft-rotated for 1 min using a sterile glass stem, left to air-dry on a nylon-filter for 30 min (25°C) to drain the excess liquid and packed in a commercially sterile polyethylene container (with a lid). Thereafter, one group of fruits was stored at RT (25°C, RH of 85%) while the other group was stored at a LT (12°C, RH of 85%; de Sousa et al., 2013). As a control treatment, sterile distilled water containing glycerol (2 g/100 mL) were used in replacement of the CHI and CAR dispersions (dos Santos et al., 2012). Each treatment included 40 fruits and different storage times (RT: 1, 4, 8, and 12 days; LT: 1, 6, 12, 18, and 24 days). The results were expressed as the storage time for appearance of visible signs of fungal infection and as percentage of fruits that presented visible signs of fungal infection at different storage time intervals times (dos Santos et al., 2012).

### Effects on Physicochemical Parameters of Fruits

Cherry tomato fruits coated and uncoated with the different combinations of CHI and CAR were evaluated for weight loss, color, firmness, soluble solids (SSs) and titratable acidity (TA) at time 1, 4, 8, and 12 days of storage at RT and at 1, 6, 12, and 24 days of storage at LT (five fruits were analyzed in each time interval). The SS content was determined using a digital refractometer (Model HI 96801, Hanna Instruments, São Paulo, Brazil), and the results were expressed as Brix (dos Santos et al., 2012). The TA was determined using phenolphthalein as an indicator with 0.1N NaOH, and the results were expressed as mmol H^+^/100 g of fruit (dos Santos et al., 2012). The weight loss of fruits was measured by monitoring the weight of the fruit at different storage periods, and determined as a percentage of the initial weight (dos Santos et al., 2012). The skin color was determined at three different equatorial positions of the fruit using the CIE*Lab* system (L^∗^a^∗^b^∗^). Hue angle (*h^∗^_ab_*) and chroma (*C^∗^_ab_*) were determined using a CR-300 colorimeter (MINOLTA Co., Osaka, Japan) and a 10-mm quartz cuvette for the readings, as described by the International Commission on Illumination (Commission Internationale de l’Éclairage [CIE], 1986; de Sousa et al., 2013). The CIELab color scale (L^∗^a^∗^b^∗^) was used with a D^65^ illuminant (standard daylight) at a 10° angle, and the apparatus was calibrated (using reference plates) in the reflectance mode (with specular reflection excluded). The firmness was determined using a 3-mm diameter probe (1/8) coupled to a TA-XT2 Texture Analyzer (Stable Micro Systems, Haslemere, UK), and the results were expressed as N/mm (de Sousa et al., 2013).

### Statistical Analysis

The assessment of the effects on fungal growth/survival and on the physicochemical parameters of cherry tomato fruits was performed in triplicate in three different independent experiments, and the results were expressed as the mean of the data. Descriptive statistics (mean and SD) and inferential tests (ANOVA followed by the Mann–Whitney test or the Kruskal–Wallis test) were performed to determine statistically significant differences (*P* ≤ 0.05) between the treatments (coated and uncoated fruits). For statistical analysis, the computational software ORIGIN 8.0 (de Sousa et al., 2013) was used. For MIC determination assays, the results are expressed as modal values because the MIC values were the same in all repetitions.

## Results

### *In Vitro* Anti-*A. Flavus* Effects

Chitosan and CAR displayed MIC values of 7.5 mg/mL and 10 μL/mL, respectively, against *A. flavus*. CHI at 7.5 or 3.75 mg/mL and CAR at 5 and 2.5 μL/mL were tested in different combinations (CHI 7.5 mg/mL + CAR 5 μL/mL, CHI 7.5 mg/mL + CAR 2.5 μL/mL, CHI 3.75 mg/mL + CAR 5 μL/mL and CHI 3.75 mg/mL + CAR 2.5 μL/mL) in assays that measured the effects on mycelial growth and spore germination of *A. flavus*. During the 7-days incubation-period, the combined CHI and CAR concentrations strongly inhibited the mycelial growth of *A. flavus* (77.2–100%) when compared with the control assay (**Table 1**). The application of CHI and CAR at the different tested combinations also showed high rates of spore germination inhibition on *A. flavus* (86.3–100%; **Table 1**). The rates of spore germination inhibition caused by all assayed combined concentrations were as higher as 85% when compared with the number of spores germinated in the control treatment (germinated spores number near to 8.3 × 10^5^ spores/mL). The combination of CHI 7.5 mg/mL + CAR 5 μL/mL presented the highest rates (*P ≤* 0.05) of inhibition on *A. flavus* in either the mycelial growth or spore germination assay.

**Table 1:** Inhibition percentage of fungal mycelial growth and spore germination of *Aspergillus flavus* URM 4550 in liquid medium containing chitosan (CHI) from *Mucor circinelloides* UCP 050 (CHI) and carvacrol (CAR) at different concentrations.

Treatment	Mycelial growth inhibition		Spore germination inhibition
	Exposure time		
.	3 days	7 days	
CHI7.5 mg/mL + CAR5 μL/mL	88.3% (±0.0%)^Aa^	100% (±0.0%)^Ab^	100% (±0.0%)^A^
CHI7.5 mg/mL + CAR2.5 μL/mL	79.4% (±4.1%)^Ba^	95.3% (±1.5%)^Bb^	94.1% (±1.30%)^B^
CHI3.75 mg/mL + CAR5 μL/mL	78.1% (±3.9%)^Ba^	92.3% (±2.9%)^Bb^	88.4% (±3.6%)^B^
CHI3.75 mg/mL + CAR 2.5 μL/mL	77.2% (±2.3%)^Ba^	88.2% (±3.1%)^Bb^	86.3% (±2.5%)^B^

*The results expressed as percent inhibition rates (±SD) of fungal mycelial growth (dry mass) and spore germination in relation to the control assay. ^A,B^For each assay, different superscript uppercase letters in the same column denote differences (P ≤ 0.05) between the mean values according to the Kruskal–Wallis test. ^a,b^For mycelial growth inhibition assay results, different superscript uppercase letters in the same row denote differences (P ≤ 0.05) between the mean values according to the Mann–Whitney test*.

Given the strong inhibition of mycelial growth and fungal spore germination, CHI and CAR were assayed in combinations of CHI 3.75 mg/mL + CAR 2.5 μL/mL and CHI 3.75 mg/mL + CAR 1.25 μL/mL to evaluate the effects as a coating on the development of *A. flavus* infection on cherry tomato fruits, as well as the effects on physicochemical quality parameters of the fruits during storage.

### *In Situ* Antifungal Effects

When fresh cherry tomato fruits were artificially contaminated with spores of *A. flavus* and treated with the coatings composed of CHI and CAR combined at different combinations, the *A. flavus* growth was delayed during the storage at both RT and LT (**Table 2**). The tomato fruits stored at RT and coated with CHI 3.75 mg/mL + CAR 2.5 μL/mL displayed visible signs (signs) of *A. flavus* infection only at the last assessed storage time interval (12th day, 15% of fruits were infected). Fruits coated with CHI 3.75 mg/mL + CAR 1.25 μL/mL and stored at RT revealed signs of infection on the eighth day of storage (25% of fruits were infected), and 30% of the fruits were infected at the end of the assessed storage period (12th day). Fruits uncoated with CHI and CAR exhibited signs of infection as early as the fourth day of storage at RT (65% of fruits were infected), and all of these fruits displayed infection signs at the end of the storage period (**Table 2**).

**Table 2:** Mean values for incidence of *A. flavus* infection on cherry tomato fruits uncoated and coated with the combination of chitosan from *M. circinelloides* UCP 050 (CHI) and carvacrol (CAR) at different concentrations and stored at room temperature (RT; 25°C) for 12 days or at low temperature (LT; 12°C) for 24 days.

Treatment	Days of storage (RT)			
	1	4	8	12
Control^∗^	0% (±0%)^a^	65% (±3%)^b^	83% (±4%)^c^	100% (±0%)^d^
CHI 3.75 mg/mL + CAR 2.5 μL/mL	0% (±0%)^a^	0% (±0%)^a^	0% (±0%)^a^	15% (±1%)^b^
CHI 3.75 mg/mL + CAR 1.25 μL/mL	0% (±0%)^a^	0% (±0%)^a^	25% (±2%)^b^	30% (±3%)^b^

**Treatment**	**Days of storage (LT)**			
	**1**	**6**	**12**	**24**

Control^∗^	0% (±0%)	70% (±2%)^b^	78% (±3%)^c^	93% (±2%)^c^
CHI 3.75 mg/mL + CAR 2.5 μL/mL	0% (±0%)	0% (±0%)^a^	0% (±0%)^a^	0% (±0%)^a^
CHI 3.75 mg/mL + CAR 1.25 μL/mL	0% (±0%)	0% (±0%)^a^	15% (±1%)^b^	20% (±1%)^b^

*^∗^Control: CHI 0 mg/mL + CAR 0 μL/mL. ^a-d^For each trial, different superscript lowercase letters in the column denote differences (P ≤ 0.05) between the mean values according to Kruskal–Wallis test*.

Fruits coated with CHI 3.75 mg/mL + CAR 1.25 μL/mL displayed signs of infection only from the 12th day of storage at LT (15% of fruits were infected), but only 20% of the fruits were infected at the end of the assessed storage period (24th day). Fruits that were not coated with CHI and CAR and were stored at LT exhibited signs of *A. flavus* infection at the sixth day of storage (70% of fruits were infected), and at the end of the assessed storage period 93% of these fruits were infected (**Table 2**). In general, the inhibition of *A. flavus* infection in cherry tomato fruits by the combinations of CHI and CAR was weakened over time.

The application of the coatings composed of CHI and CAR also inhibited the occurrence of infection caused by native fungal population in cherry tomato fruits (**Table 3**). Fungal infection was not visible in fruits treated with the coating containing CHI 3.75 mg/mL + CAR 2.5 μL/mL or CHI 3.75 mg/mL + CAR 1.25 μL/mL during the assessed storage intervals at LT. The fruits treated with coating containing CHI 3.75 mg/mL + CAR 1.25 μL/mL displayed signs of fungal infection from the fourth day of storage at RT (15% of fruits were infected), and 25% of these fruits were infected at the end of the assessed storage period. Fruits treated with the coating containing CHI 3.75 mg/mL + CAR 2.5 μL/mL and stored at RT only displayed signs of fungal infection (10% of fruits were infected) at the last assessed storage interval (12th day). Cherry tomato fruits not treated with the coatings containing CHI and CAR showed signs of fungal infection at the 4th (65% of fruits were infected) and 12th (62% of fruits were infected) days when stored at RT and LT, respectively. In general, the antifungal effects of the coatings containing CHI and CAR occurred in a dosage-dependent manner in both *in vitro* and *in situ* assays.

**Table 3:** Mean values for incidence of visible signs of fungal infection (caused by native fungal microbiota) on cherry tomato fruits uncoated and coated with the combination of chitosan from *M. circinelloides* UCP 050 (CHI) and carvacrol (CAR) at different concentrations, and stored at room temperature (RT; 25°C) for 12 days or at low temperature (LT; 12°C) for 24 days.

Treatment	Days of storage (RT)			
	1	4	8	12
Control^∗^	0% (±0%)	65% (±2%)^b^	73% (±2%)^c^	100% (±0%)^c^
CHI 3.75 mg/mL + CAR 2.5 μL/mL	0% (±0%)	0% (±0%)^a^	0% (±0%)^a^	10% (1%)^a^
CHI 3.75 mg/mL + CAR 1.25 μL/mL	0% (±0%)	15% (±1%)^b^	20% (±2%)^b^	25% (±1%)^b^

**Treatment**	**Days of storage (LT)**			
	**1**	**6**	**12**	**24**

Control^∗^	0% (±0%)	0% (±0%)	62% (±3%)^b^	86% (±2%)^b^
CHI 3.75 mg/mL + CAR 2.5 μL/mL	0% (±0%)	0% (±0%)	0% (±0%)^a^	0% (±0%)^a^
CHI 3.75 mg/mL + CAR 1.25 μL/mL	0% (±0%)	0% (±0%)	0% (±0%)^a^	0% (±0%)^a^

*^∗^Control: CHI 0 mg/mL + CAR: 0 μL/mL. ^a-c^For each trial, different superscript lowercase letters in the same column denote differences (P ≤ 0.05) between the mean values according to Kruskal–Wallis test*.

### Effects On Physicochemical Characteristics of Fruits

Some physicochemical parameters in fresh cherry tomato fruits uncoated or coated with CHI3.75 mg/mL + CAR 2.5 μL/mL or CHI 3.75 mg/mL + CAR 1.25 μL/mL were evaluated during storage (**Table 4**). For most of them, no differences (*P* > 0.05) were observed among the coated and non-coated fruits during the assessed storage periods at both RT and LT.

**Table 4:** Mean values for weight loss and some physicochemical quality parameters in cherry tomato fruits uncoated and coated with the combination of CHI from *M. circinelloides* UCP 050 (CHI) and carvacrol (CAR) at different concentrations and stored at room temperature (RT; 25°C) for 12 days or at low temperature (LT; 12°C) for 24 days.

Treatment	Days of storage (RT)			
	1	4	8	12
**Weight loss (%)**				
Control^∗^	3.1% (±0.3)^b^	10.3% (±1.5)^c^	21.4% (±2.7)^b^	25.2% (±2.7)^b^
CHI 3.75 mg/mL + CAR 2.5 μL/mL	0.0% (±0.0)^a^	6.5% (±1.2)^b^	12.1% (±1.8)^a^	14.1% (±1.9)^a^
CHI 3.75 mg/mL + CAR 1.25 μL/mL	0.0% (±0.0)^a^	4.3% (±0.9)^a^	10.1% (±1.3)^a^	11.1% (±1.1)^a^
**Firmness (N/mm)**				
Control	4.9 (±0.8)	3.3 (±0.8)	3.6 (±0.5)	3.5 (±0.74)
CHI 3.75 mg/mL + CAR 2.5 μL/mL	5.1 (±0.9)	4.6 (±0.9)	3.9 (±0.9)	3.9 (±0.75)
CHI 3.75 mg/mL + CAR 1.25 μL/mL	5.1 (±0.9)	3.7 (±0.9)	3.6 (±0.8)	3.4 (±0.78)
**Titratable acidity (mmol H^+^/100 g of fruit)**				
Control^∗^	0.4 (±0.1)^a^	0.32 (±0.0)^a^	0.2 (±0.1)^a^	0.20 (±0.0)^a^
CHI 3.75 mg/mL + CAR 2.5 μL/mL	0.4 (±0.1)^b^	0.40 (±0.0)^b^	0.3 (±0.0)^b^	0.30 (±0.0)^b^
CHI 3.75 mg/mL + CAR 1.25 μL/mL	0.4 (±0.2)^b^	0.40 (±0.0)^b^	0.3 (±0.0)^b^	0.29 (±0.1)^b^
**Soluble solids (°Brix)**				
Control^∗^	4.7 (±0.3)	5.0 (±0.3)^b^	5.2 (±0.3)^b^	5.36 (±0.4)^b^
CHI 3.75 mg/mL + CAR 2.5 μL/mL	4.3 (±0.4)	4.0 (±0.2)^a^	4.2 (±0.2)^a^	4.43 (±0.3)^a^
CHI 3.75 mg/mL + CAR 1.25 μL/mL	4.0 (±0.3)	3.8 (±0.4)^a^	4.2 (± 0.3)^a^	4.52 (± 0.7)^a^

**Treatment**	**Days of Storage (LT)**			
	**1**	**6**	**12**	**24**

**Weight loss (%)**				
Control^∗^	0.0% (±0.0)	6.4% (±1.2)^c^	15.0% (±1.6)^b^	21.3% (± 2.2)^c^
CHI 3.75 mg/mL + CAR 2.5 μL/mL	0.0% (±0.0)	2.1% (±0.4)^b^	5.1% (±0.4)^a^	7.1% (± 0.6)^b^
CHI 3.75 mg/mL + CAR 1.25 μL/mL	0.0% (±0.0)	3.4% (±0.4)^a^	6.1% (±0.5)^a^	10.1% (± 1.5)^a^
**Firmness (N/mm)**				
Control^∗^	5.3 (±0.5)	4.6 (±0.3)	4.1 (±0.6)	3.3 (± 0.6)
CHI 3.75 mg/mL + CAR 2.5 μL/mL	5.4 (±0.4)	4.7 (±0.8)	4.1 (±0.5)	3.6 (± 0.4)
CHI 3.75 mg/mL + CAR 1.25 μL/mL	5.8 (±0.3)	4.9 (±0.8)	4.2 (±0.7)	3.4 (± 0.5)
**Titratable acidity (mmol H+/100 g of fruit)**				
Control^∗^	0.5 (±0.0)	0.4 (±0.0)	0.3 (±0.0)	0.3 (±0.1)
CHI 3.75 mg/mL + CAR 2.5 μL/mL	0.5 (±0.4)	0.4 (±0.0)	0.3 (±0.1)	0.3 (± 0.0)
CHI 3.75 mg/mL + CAR 1.25 μL/mL	0.4 (±0.6)	0.4 (±0.1)	0.4 (±0.1)	0.3 (±0.0)
**Soluble solids (°Brix)**				
Control^∗^	4.1 (±0.4)	4.0 (±0.8)	4.8 (±0.3)	5.1 (±0.6)
CHI 3.75 mg/mL + CAR 2.5 μL/mL	4.3 (±0.2)	4.3 (±0.6)	4.8 (±0.6)	5.2 (±0.6)
CHI 3.75 mg/mL + CAR 1.25 μL/mL	4.4 (±0.2)	4.4 (±0.7)	4.9 (± 0.5)	5.1 (± 0.7)

*^∗^Control: CHI 0 mg/mL + CAR: 0 μL/mL. ^a-c^For each trial, different superscript lowercase letters in the same column denote differences (P ≤ 0.05) between the mean values according to Kruskal–Wallis test*.

Fruits coated or uncoated with the combinations of CHI and CAR differed (*P* ≤ 0.05) in weight loss at the end of the storage time, although the weight loss of all fruits groups increased gradually during storage. Fruits coated with CHI 3.75 mg/mL + CAR 2.5 μL/mL or CHI 3.75 mg/mL + CAR 1.25 μL/mL and stored at RT exhibited weight losses of 14.2 and 11.1%, respectively, at the end of the assessed storage time interval. The weight losses of the tomato fruits coated with either the combination of CHI 3.75 mg/mL + CAR 2.5 μL/mL or CHI 3.75 mg/mL + CAR 1.25 μL/mL and stored at LT were 7.1 and 10.1%, respectively, at the end of the assessed storage period. The uncoated fruits stored at RT and LT displayed weight loss of 25.2 and 21.3% at the end of the assessed storage period.

No difference (*P* > 0.05) in firmness was observed among coated and uncoated fruits during the storage at LT. Both the coated and uncoated fruits presented a sharp decrease (*P* ≤ 0.05) in firmness during the storage period at both tested storage temperatures. Otherwise, cherry tomato fruits coated with the combinations of CHI and CAR presented higher (*P* ≤ 0.05) values of TA than the uncoated fruits when stored at RT. Moreover, uncoated fruits displayed higher (*P* ≤ 0.05) values of SS than the coated fruits from the 4th day of storage at RT onward. Cherry tomato fruits coated or uncoated with combinations of CHI and CAR and stored at LT did not differ (*P* > 0.05) in any of the studied physicochemical parameters at all assessed time intervals, with exception of weight loss from the sixth day of storage onward.

The cherry tomato fruits uncoated and coated with CHI 3.75 mg/mL + CAR 2.5 L/mL or CHI 3.75 mg/mL + CAR 1.25 μL/mL were predominantly red throughout storage at both tested storage temperatures. The fruits submitted to all treatment conditions displayed an increase (*P* ≤ 0.05) in *a*^∗^ and *b*^∗^ coordinates, resulting in a lightness decrease because of the enhancement in fruit opacity. For coated and uncoated fruits, there was a decrease (*P* ≤ 0.05) in *h^∗^* values and maintenance (*P* > 0.05) of *C^∗^* values during storage, showing a color change from red to dark red. Still, both coated and uncoated fruits also displayed similar (*P* > 0.05) L^∗^ values during storage, showing the maintenance of brightness (data not shown).

Taken together, these results demonstrate that combinations of CHI and CAR at subinhibitory concentrations effectively reduce fungal decay and maintain the quality of fresh cherry tomato fruits over storage.

## Discussion

The combined application of CHI and CAR at different concentrations (MIC values and/or subinhibitory concentrations) induced the inhibition of the mycelial growth and spore germination of *A. flavus* in laboratory media. These findings are interesting because fungi are commonly less tolerant to antimicrobial compounds during the mycelial growth stages than during the spore germination stage, most likely because the stronger antimicrobial resistance of fungal spore structures (de Oliveira et al., 2014a,b). The strong and fast inhibition of *A. flavus* spore germination induced by CHI and CAR is worthy of note considering their practical application on fruits because the spores are important structures in the survival and spread of pathogenic fungi in horticultural products, causing the fruits infections, rot development and postharvest losses (de Sousa et al., 2013).

The combinations of CHI and CAR also presented strong antifungal effects when it was applied forming different coatings on cherry tomato fruits artificially contaminated with *A. flavus* spores, as well as against the native mycoflora of these fruits during storage at RT or LT. The CHI concentrations of 7 and 3.75 mg/mL were used to form the tested coatings because 3.75 mg/mL was the lowest CHI concentration that was capable of forming a viscous solution that permitted its application as a coating for grapes. Still, CHI at 10 mg/mL formed highly viscous dispersions that did permit the formation of continuous films when they were applied to fruits. For CAR, the concentrations were set at 5 and 2.5 μL/mL because researchers have stated that essential oils or their individual constituents could be incorporated at low concentrations (<10 μL/mL) in coating dispersions to minimize their possible impact on the olfactory perception of consumers (Perdones et al., 2012).

An early study found that the combination of crustacean CHI and *Origanum vulagre* L. essential oil (OVEO), presenting CAR as the major compound (66.9 g/100 mL), at subinhibitory amounts caused high inhibition rates of spores germination and morphological changes in spores (wilting, disruption, loss of cellular material and deepening of ridges) of *Aspergillus niger* and *Rhizopus stolonifer* (dos Santos et al., 2012). The authors also detected decreased occurrence of infections caused by *A. niger* and *R. stolonifer* in artificially contaminated table grapes that were treated with the combinations of CHI and OVEO (dos Santos et al., 2012). The mechanism by which CHI and OVEO induce the inhibition of spore germination has been related with the interaction of CHI and the essential oil individual constituents, mainly the major compound CAR, with the spore cell wall. The negative effect of CAR on enzymatic reactions needed for the synthesis of fungal spore cell wall components is also related with its effects on spores germination and viability and ultimately the fungal growth (dos Santos et al., 2012; de Sousa et al., 2013).

The effects of the coating comprising the combinations of CHI and CAR in delaying the appearance of *A. flavus* infection in cherry tomato fruits increased when the fruits were stored at LT. Researchers have proposed that storage at LT slows physiological processes in fruits and delay the senescence, becoming the fruits more resistant to fungal diseases. Moreover, it has been proposed that most of the postharvest pathogen fungi have weaker pathogenicity at LT, resulting in a decreased incidence of fungal infections and decay compared to fruit stored at RT (de Oliveira et al., 2014a,b).

The application of the coatings containing CHI and CAR did not negatively affected the physicochemical characteristics of cherry tomato fruits throughout the storage period, as assessed by weight loss, firmness, TA value, and SS content. Tomato fruits coated with the combinations of CHI and CAR lost less weight during storage at either RT or LT. Weight loss in fresh fruits is primarily associated with the water loss caused by transpiration and respiration (Meng et al., 2008). Decreased weight loss in cherry tomato fruits coated with CHI and CAR is associated with the formation of a protective barrier that reduces respiration and transpiration across the fruit surface (Liu et al., 2007; Gol et al., 2013). Because of its hydrophobic properties, the incorporation of CAR into CHI-based coatings may improve the preventive effects and reduce moisture loss in coated cherry tomato fruits (Vargas et al., 2008).

The coatings composed of CHI and CAR induced a decrease in the SS contents of cherry tomato fruits stored at RT. This decrease could be associated with the slowing of respiration and metabolic activity in the fruit, hence delaying the ripening process (Hong et al., 2012). CHI-based dispersions are capable of forming a semi-permeable barrier when applied as a coating in fruits, reducing O_2_ and/or elevating CO_2_ and suppressing ethylene production (Dong et al., 2004; de Oliveira et al., 2014a). This suppressed respiration rate slows down the metabolic activity, resulting in lower levels of SS because the slower hydrolysis of carbohydrates to sugars (Hong et al., 2012; Gol et al., 2013; de Oliveira et al., 2014a). Previous studies also found that the application of coatings composed of CHI, alone or in combination with essential oil individual constituents, decreased the amount of SS in different fruits (Kitur et al., 2001; Ali et al., 2011; dos Santos et al., 2012; de Oliveira et al., 2014b), and these changes were most pronounced in RT-stored fruits.

Although the impact of the composite coatings containing CHI and CAR on sensory characteristics of cherry tomato fruits has not been assessed, previous studies found that the application of essential oils containing CAR as major constituent (>65/100 g) alone or combined with CHI do not impact negatively the sensory acceptance and purchase intention of fruits (dos Santos et al., 2012; de Sousa et al., 2013).

The results obtained in this study revealed that the application of coatings composed of CHI from *M. circinelloides* and CAR at different subinhibitory concentrations can significantly inhibit the postharvest pathogenic fungus *A. flavus*, as well as the native fungal microbiota, on fresh cherry tomato fruits during storage at RT and LT. However, these inhibitory effects were more pronounced when the fruits were stored at LT. Moreover, the tested coatings displayed no negative influence on the physicochemical aspects of cherry tomato fruits during the storage. These findings demonstrate the potential for using CHI from *M. circinelloides* and CAR for the formulation of coatings to be applied in the control of pathogenic fungi in fruits, particularly *A. flavus* on cherry tomato fruits, which may be non-chemical alternatives to improve the quality of these commodities and provide greater economic benefits to producers.

## Conflict of Interest Statement

The authors declare that the research was conducted in the absence of any commercial or financial relationships that could be construed as a potential conflict of interest.
